# CAR T-Cell Therapy Is Effective but Not Long-Lasting in B-Cell Lymphoma of the Brain

**DOI:** 10.3389/fonc.2020.01306

**Published:** 2020-08-04

**Authors:** Tongjuan Li, Lei Zhao, Yuanyuan Zhang, Yi Xiao, Di Wang, Liang Huang, Liya Ma, Liting Chen, Songya Liu, Xiaolu Long, Fankai Meng, Xiaojian Zhu, Jia Wei, Bin Xu, Jianfeng Zhou, Xiaoxi Zhou

**Affiliations:** ^1^Department of Hematology, Tongji Hospital, Tongji Medical College, Huazhong University of Science and Technology, Wuhan, China; ^2^Department of Radiology, Tongji Hospital, Tongji Medical College, Huazhong University of Science and Technology, Wuhan, China

**Keywords:** CNS lymphoma, CAR T-cell therapy, CRES, recurrence, tumor immunosuppressive microenvironment

## Abstract

Advanced central nervous system (CNS) lymphoma is an exclusion criterion for most chimeric antigen receptor (CAR) T-cell studies due to the associated levels of neurotoxicity. In this study, we described five patients with chemorefractory B-cell CNS lymphoma who received CAR19 and CAR22 T-cell “Cocktail” therapy and follow-up for 6–16 months. All patients experienced cytokine release syndrome (CRS). Two patients experienced CAR T-cell-related encephalopathy syndrome (CRES), which was controllable. The best response was observed in two patients, who successfully achieved complete remissions (CR), and the other three patients achieved partial remissions (PR). Four patients had progressive disease (PD) after remission. In addition, one CR patient and one PD patient accepted CAR T-cell infusion following hematopoietic stem cell transplantation therapy in the 3rd month and were in ongoing remission for 14 and 6 months of follow-up, respectively. The targeted antigens in two patients were still positive, and CAR T-cells were reboosted in the cerebrospinal fluid (CSF) after PD, but a small number of CD3-positive T-cells were observed to infiltrate into the tumor. Our study indicates the efficacy of CAR T-cell therapy for CNS lymphoma with an acceptable safety profile; however, the remission did not last long, perhaps due to the tumor immunosuppressive microenvironment (TME) of the CNS. CAR T-cell therapy should be combined with other treatments to help improve the TME of cerebral lymphoma.

## Introduction

The incidence of central nervous system (CNS) relapse among diffuse large B-cell lymphoma (DLBCL) patients is ~4% (1, 2). For B-cell CNS lymphoma, current therapies, such as high-dose chemotherapy, radiation, and some targeted therapy drugs have shown little success in improving very poor patient outcomes. A variety of studies have shown that the blood-brain barrier (BBB) limits the efficacy of many drugs, including antibody therapy drugs such as rituximab (3, 4). Although intensive chemotherapy followed by hematopoietic stem-cell rescue has achieved good results in chemosensitive patients, the outcomes of chemorefractory patients are still in question, especially the outcomes of patients with brain parenchymal lymphoma involvement worse than those of patients with isolated intraocular lymphoma and/or leptomeningeal involvement (5, 6). In addition, these therapies are associated with an increased risk of treatment-related complications and morbidity. For example, one clinical trial showed that patients ≥60 years old who received both HD-MTX and whole-brain radiotherapy had a 100% incidence of neurotoxicity within 2 years of treatment (7).

Chimeric antigen receptor (CAR) T-cell therapy is a promising immunotherapy for treating relapsed/refractory B-cell leukemia and lymphoma. Studies of anti-CD19 CAR T-cells in refractory DLBCL have shown exciting results, with 50% complete remission (CR) and high rates of durable remission (8–10). However, due to concerns about neurotoxicity, advanced CNS disease is an exclusion criterion for most CAR T-cell studies (9–11). Recently, a report presented a patient with refractory secondary CNS DLBCL who received CD19 CAR T-cell treatment and achieved CR without cytokine release syndrome (CRS) or neurotoxic effects (12). Another report presented eight secondary CNS lymphoma patients who received tisagenlecleucel CAR T-cell therapy, none of whom experienced > grade 1 neurotoxicity (13). However, the brain is an organ with immune privilege, and its response persistence to CAR T-cell therapy is unknown.

Here, we report five cases of CAR T-cell therapy for chemorefractory B-cell parenchymal lymphoma of the brain. We demonstrate that CAR T-cell therapy is safe and feasible for the treatment of CNS lymphoma. However, the remission observed did not last long, perhaps due to composition of the tumor immunosuppressive microenvironment (TME) of the CNS changing under CAR T-cell therapy stress.

## Methods

Five B-cell CNS lymphoma patients were enrolled in this study [third-generation CAR (CAR19/22) T-cell “cocktail” in patients with relapse/refractory B-cell malignancies, registry number ChiCTR-OPN-16008526 at http://www.chictr.org.cn]. Four Patients had secondary CNS lymphomas, and one had primary CNS lymphoma (patient 5, PCNSL). The study was approved by the Ethics Committee of Tongji Hospital, Tongji Medical College, Huazhong University of Science and Technology, and all subjects provided written informed consent.

The CD19 and CD22 CAR T-cell manufacturing processes were previously described (14). The treatment response was assessed monthly for the first 6 months after infusion and then at every 2 months. CRS was evaluated using the modified criteria of Lee et al. (15). CRES was graded according to the National Cancer Institute CTCAE (Version 5.0) (16, 17).

Flow cytometry was used to detect CAR T-cells in peripheral blood (PB) and CSF. The CAR gene-specific MGB probe was designed by Primer Express 3.0. Absolute quantification of the CAR gene copy numbers was determined by droplet digital polymerase chain reaction (ddPCR) before and after infusion of CAR T-cells.

Fluorescence *in situ* hybridization (FISH) was performed to detect the amplification/translocation of *MYC, BCL2*, and *BCL6*. HiSeq deep sequencing of 173 lymphoma-related genes with mutation hot spots was performed initially with an Ion Torrent PGM/Illumina NextSeq 550Dx platform and verified with an ABI3500 platform.

## Results

### Patient Characteristics

The clinical characteristics and treatments of the five patients (three males and two females, 18–60 years old) are listed in Tables 1, 2, respectively. Four patients were diagnosed with stage IV DLBCL with secondary CNS involvement. One patient was diagnosed with PCNSL (pathology, DLBCL). The age-adjusted International Prognostic Index (aaIPI) score of all patients was 3. Symptoms and signs of headache, muscle weakness of the lower limbs, deviated mouth, and dysphagia were observed in the patients. The Karnofsky performance Status (KPS) score ranged from 20 to 50. Some cases had high-risk genetic factors. FISH examinations for *BCL2, BCL6*, and *MYC* were performed for four patients. Among the four patients, two had a positive result, including *MYC* rearrangement and *MYC* amplification separately in patients 2 and 5. HiSeq deep sequencing was performed for four patients. The specimens were derived from PB (patients 1, 2, and 3) and tumor tissue (patient 5). Three out of four patients had positive findings, including an *ATM* mutation in patient 1, *MLL2, CREBBP*, and *ID3* mutations in patient 2, and *MYD88* and *PIM1* mutations in patient 5. All patients had brain parenchyma lesions, and the locations of the tumor sites within the brain varied from patient to patient. Lymphoma invasion of the meninges and systemic lesions was found in patients 2 and 3, respectively. The clinical features are summarized in Table 1. The lesions assessments in Table 1 were performed after the chemotherapy during the waiting time for CAR-T cell preparation.

**Table 1:** Clinical characteristics of patients during the time of CAR-T cells preparation.

**ID**	**Sex**	**Age**	**KPS**	**Disease pathology**	**Stage**	**aaIPI**	***^a^*HiSeq deep sequencing**	**FISH**	**Site of CNS Disease (maximal dimension)**	**Tumor in CSF (%)**	**Systemic Disease**
1	M	39	50	DLBCL non-GCB	IV	3	Negative	^*^NA	Left cerebellum (4.2 cm), corpus callosum (1.8 cm)	0	None
2	M	60	40	DLBCL GCB	IV	3	*ATM*	*MYC* rearrangement	Bilateral frontal cortex, white matter, thalamus (1.4 cm)	60	None
3	M	38	20	DLBCL GCB	IV	3	*MLL2, CREBBP, GNA13, ID3*	Negative	Left lateral ventricle (1.9cm)	0	Right ilium, iliacus, gluteus
4	F	18	40	DLBCL NOS	IV	3	^*^NA	Negative	Right basal ganglia, insula, frontal parietal temporal white matter, radiating crown, lateral ventricular trigonometry (2.8 cm)	0	None
5	F	49	20	DLBCL GCB	IV	3	*MYD88, BTG1, PIM1, TBL1XR1*	*MYC* amplification	Left occipital parietal lobe ventricle (4.6 cm)	0	None

*M, male; F, female; KPS, Karnofsky Performance Status; aaIPI, age-adjusted International Prognostic Index; DLBCL, diffuse large B cell lymphoma; GCB, germinal center (GC)-like B-cell type; NOS, not otherwise specified*.

a ATM frameshift code deletion in exon 47 (c.6815del, p. R2273Gfs*37); MLL2 mutation in splicing point (c.6183+2T>C); CREBBP frameshift code deletion in exon 5 (c.1222 del, p.H408Ifs*); GNA13 splicing point mutation (c.510+2T>A, p.A161V); ID3 missense mutation in exon 1 (c.117C>G, p.S39R); MYD88 missense mutation in exon 5(c.794T>C, p. Leu265Pro); BTG1 nonsense mutation in exon 2 (c.168G>A, p. Trp56*); PIM1 missense mutation in exon 4 (c.241C>T, p. Pro81Ser); TBL1XR1 frameshift code insertion in exon 10(c868dup, p. Thr290Asnfs*10).

* *NA, not available*.

*FISH, fluorescence in situ hybridization; CSF, cerebrospinal fluid; Tumor in CSF (%), percentage of B lymphoma cells in nuclear cells of CSF*.

**Table 2:** Treatment and effect of CAR-T cell therapy.

**ID**	**Primary treatment**	**Before CAR-T treatment**	**CAR-T treatment**						**Follow-up Treatment**
			**Infused cells (10^**6**^/kg)**	**CRS grade**	**CRES grade**	**Neurologic toxicity**	**CRS/CRES Treatment**	**Time to best response (days)**	
1	- R-CHOP×6 (CR) **Isolated CNS relapse** - HD-MTX+R+ temozolomide (PD)	HD-MTX Temozolomide	CD22 7.0 CD19 3.0	1	0	None	None	30	Radiotherapy×7F, PD-1 inhibitor×3
2	- Radiotherapy×13F (SD) - R-CHOP×8 (CR) - Lenalidomide **Isolated CNS relapse** - R-ICE×3 (PD) - R-MA×3 (PD)	CYVE Temozolomide Ibrutinib	CD22 3.12 CD19 2.23	1	1	Transient decreased mathematics ability, maxillofacial numbness	None	60	Radiotherapy×16F
3	- R-DA-EPOCH×2 (PD) - R-HD-MTX (PD) - R-DHAP (PD) - R-HD-Ara-C×2 (PD) **Systemic disease progression and CNS involvement**	Liposomal Doxorubicin Decitabine Dexamethasone	CD22 4.54 CD19 7.06	2	4	Somnolent, delirium, abnormal behavior, dysmnesia	Plasmapheresis, tocilizumab, glucocorticoid	60	CAR-T combined with ASCT
4	- R-CHOP×5 (CR) - R-DA-EPOCH - R-HD-MTX, R **Isolated CNS relapse**	R-HD-MTXRadiotherapy×5F	CD22 6.0 CD19 6.0	1	0	None	None	30	Radiotherapy
5	- HD-MTX×3 (PR) - R-HD-MTX×2 (PR then PD)	R-HD-MTX Temozolomide Lenalidomide Ibrutinib	CD22 6.0 CD19 6.0	1	0	None	None	30	CAR-T combined with ASCT

*CRS, cytokine-release syndrome; CRES, CAR-T-cell-related encephalopathy syndrome; R, rituximab; CHOP, cyclophosphamide, Vindesin, liposomal adriamycin, dexamethasone; ICE, ifosfamide, carboplatin, etoposide; MA, methotrexate, cytarabine; DA-EPOCH, vindesine, pirarubicin, etoposide, dexamethasone, cyclophosphamid; HD-MTX, high dose methotrexate; DHAP, dexamethasone, cytarabine, cisplatinum, G-CSF; CYVE, etoposide, cytarabine; HSCT, hematopoietic stem cell transplantation; PD-1 inhibitor, nivolumab*.

The patients had received multiline treatment, including chemotherapy, immunotherapy, and radiotherapy. The median number of prior therapies was 7 (range 5–15; Table 2). The disease status of all the patients before CAR T-cell infusion was classified as progressive disease (PD).

### CAR T-Cell Infusion

In view of the patients' poor clinical status, the five patients were enrolled in a clinical trial for a CAR (CAR19/22) T-cell “cocktail” therapy. During the preparation time for the CAR T-cells, the patients' symptoms and tumor progression were controlled with therapy including temozolomide, liposomal doxorubicin, decitabine, dexamethasone, high-dose methotrexate (HD-MTX) and CYVE (Table 2).

For lymphodepletion, patients received a daily FC regimen (fludarabine 25 mg/m^2^, cyclophosphamide 20 mg/kg) for 3 days before CAR T-cell infusion. Depending on the total number of cultured CAR T-cells for each patient, the number of anti-CD22 CAR T-cells infused ranged from 3.1 × 10^6^ to 7.0 × 10^6^/kg, and the number of anti-CD19 CAR T-cells infused ranged from 2.23 × 10^6^ to 7.06 × 10^6^/kg (Table 2). Third-generation anti-CD19 and anti-CD22 CAR T-cells were given separately and intravenously over 2–3 days (detail shown in Supplementary Table 1).

### Toxicities

The two most frequently observed adverse events for CAR T-cell therapy were CRS and CRES (Table 2). All patients experienced low-grade CRS. Only patient 3 experienced grade 2 CRS, and the others experienced grade 1 CRS. Two out of five patients had CRES. Grade 1 CRES was observed in patient 2. He had decreased mathematics ability and maxillofacial numbness 5 days after CAR T-cells infusion, and the symptoms lasted for 4 days without intervention. Grade 4 CRES was seen in patient 3. He had a severely high fever on day 2 that lasted for 5 days, followed by extreme drowsiness that continued for 4 days, then delirium, abnormal behavior and dysmnesia for 5 days. The CRES in this patient was well-controlled after glucocorticoid, plasmapheresis, and tocilizumab treatments. No instances of grade 5 neurotoxic events or cerebral edema was observed in the patient cohort, and the neurologic toxicity-related symptoms were completely reversible.

**Figure 1:**
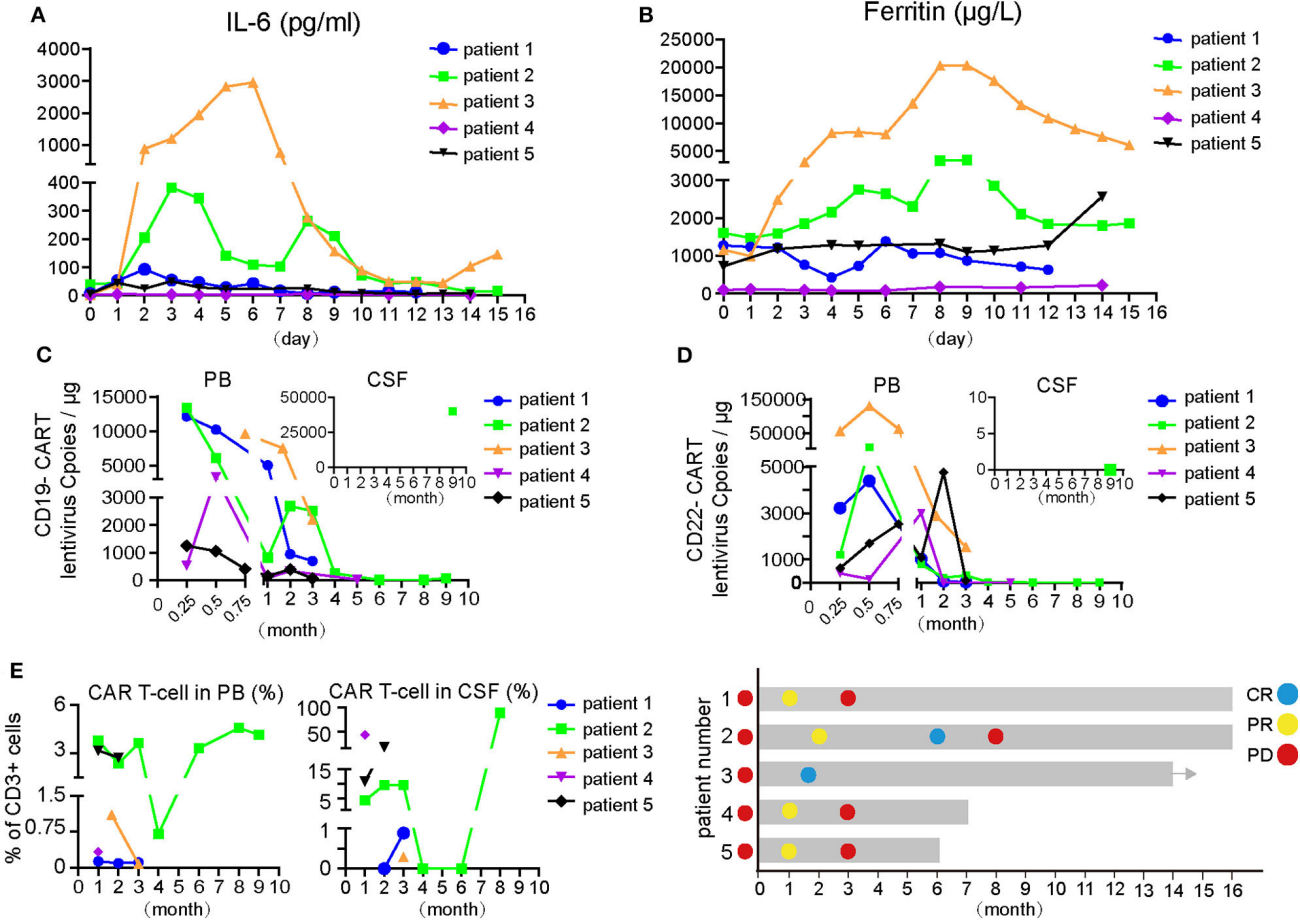
Changes of indicators during CAR-T cell therapy and therapeutic effect of CAR T treatment. **(A,B)** IL-6 and ferritin level of the five patients during CAR-T cell therapy. The day of first CAR T-cells infusion was as day 0. **(C)** Dynamic changes of lentivirus copies of CD19- or CD22-CAR-T cells in PB and partial in CSF after CAR-T cell therapy. Expansion of anti-CD19 or CD22 CAR-T cells *in vivo* were quantified by ddPCR at multiple time points after infusion. **(D)** Changes of total CART percent of the 5 patients in PB and CSF, respectively, after CAR-T cell therapy. The proportion of CAR-T cells in CD3 positive T cells was detected by flow cytometry. **(E)** Therapeutic effect and disease status of the five patients. Different colored dot represents the disease status, on the left of the *Y*-axis is the disease status before CAR-T cell therapy and right is on behalf of dynamic disease status after CAR-T cell therapy. Arrows indicate the ongoing status until now. CSF, cerebrospinal fluid; CR, complete remission; PR, partial remission; PD, progressive disease.

Increases and decreases in serum IL-6 and ferritin temporally coincided with the onset and resolution of CRS/CRES, respectively (Figures 1A,B). Patient 3 had the highest grade of CRES accompanied by the highest levels of serum IL-6 and ferritin. IL-6 and ferritin levels peaked at a median of 3 and 9 days after CAR T-cells infusion, with a median value of 92.8 pg/ml and 2,574 μg/L, respectively.

### Response To Therapy

**Figure 2:**
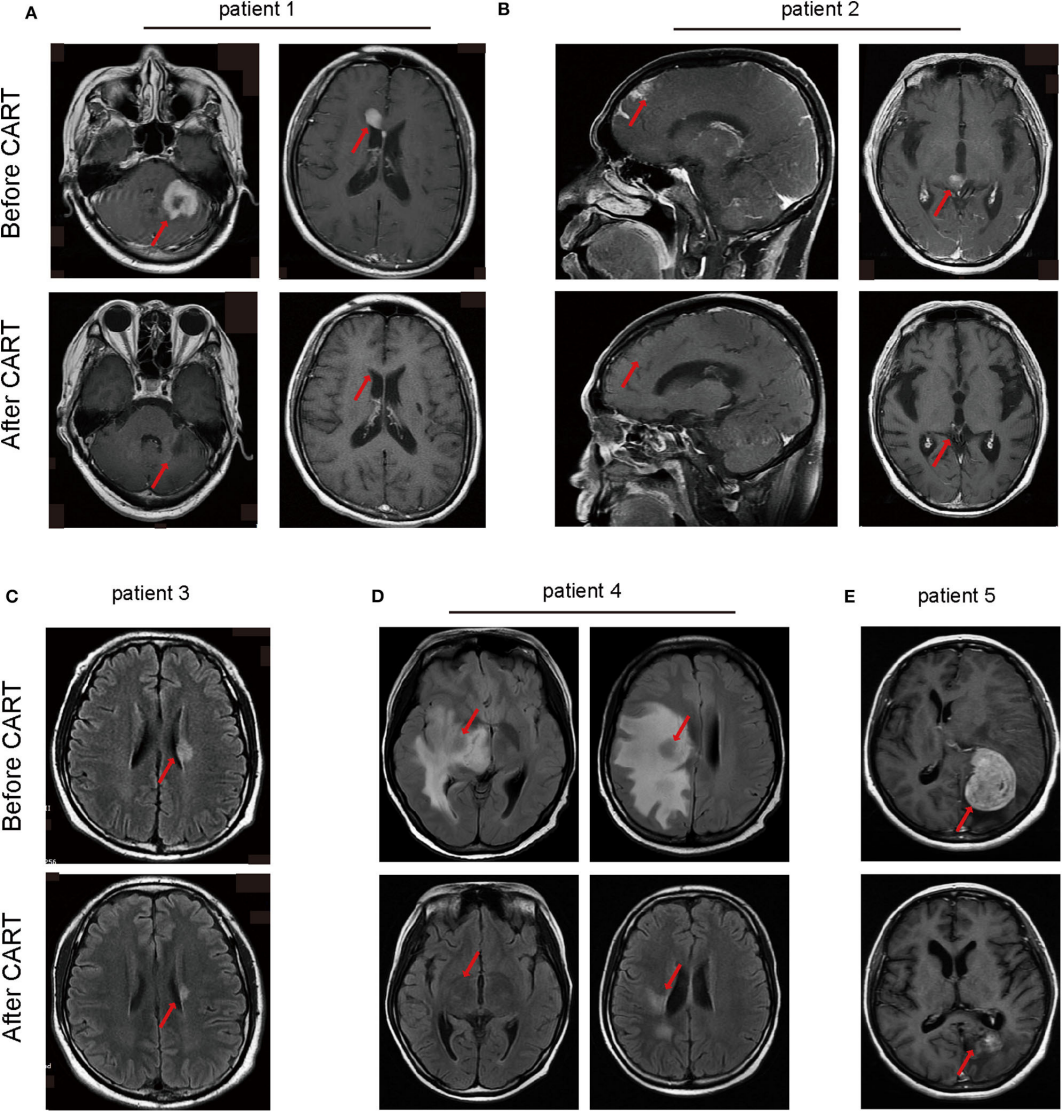
Head MRI imaging manifestations of lymphoma in CNS monitoring the therapeutic effects of CAR-T cell therapy of the five patients. **(A)** The main central invasion sites of patient 1 are left cerebellum and corpus callosum. **(B)** Invasion sites of patient 2 are frontal cortex and thalamus. **(C)** The central invasion site of patient 3 is left lateral ventricle. **(D)** The main central invasion sites of patient 4 are right basal ganglia, insula, frontal parietal temporal white matter, radiating crown, lateral ventricular trigonometry. **(E)** The invasion site of patient 5 is left occipital parietal lobe. MRI, magnetic resonance imaging.

An objective response was observed in all five patients in the 1st month after CAR T-cell therapy including obvious shrinkage of CNS lesions, and minimal residual disease (MRD) becoming negative in cerebrospinal fluid for patient 2. Among the five patients, one (patient 3) achieved CR, confirmed by PET/CT showing no metabolism of both systemic and CNS lesions, and the other four achieved PR, at the 2nd month. The CR patient (patient 3) was enrolled into another clinical trial at the 3rd month that involved sequential infusions of anti-CD22 and anti-CD19 CAR T-cells following HSCT therapy and was in ongoing remission at the most recent follow-up (14 months). One of PR patients (patient 2) had a CR showed by subsequent MRI at the 6th month but relapsed at the 8th month. The remaining three PR patients had progressive disease at the 3rd month. Of the four PD patients, patient 5 was enrolled in the same clinical trial as patient 3 and was in ongoing CR for 6 months of follow-up. The other three patients received radiotherapy and/or anti-PD1 antibody therapy and maintained stable disease for 16 months (patient 1 and 2) and 7 months (patient 4) of follow-up. The clinical outcome and subsequent treatments are listed in Table 2 and shown in Figure 1E. Heat MRI imaging findings before CAR T-cell infusion and at the time of best response are shown in Figure 2 (lesions indicated by red arrows). The overall survival was 100% in 6–16 months of follow-up, and the median progression free survival time was 3 months (Supplementary Figures 1A,B).

### CAR T-Cell Kinetics

To detect the expansion and persistence of CAR T-cells *in vivo*, we measured the number of copies of CAR in the PB and CSF and the percentage of CAR T-cells among CD3^+^ T cells in the PB and CSF. The number of CAR19 and CAR22 copies in the blood peaked around days 7–14 after the first infusion of CAR T-cells, respectively (Figure 1C). Specifically, the copy numbers of both CAR19 and CAR22 were 688.3 and 23.6 for patient 1 and 55 and 93 for patient 5, respectively, at the time of PD. For patients 2 and 4, although the number of CAR copies dropped below the limit of detection, the percentage of CD19^+^ B cells in lymphocyte was 0.02% in the 8th month and 0.05% in the 6th month, respectively. B cell aplasia persisted for more than 6 months, suggesting the possibility that CAR T-cells may have existed in the patients.

When the amount of CSF specimens collected was sufficient for analysis, detection of CAR T-cells in the CSF was performed. The available data showed that CAR T-cells were present in the CSF (Figure 1D), indicating that CAR T-cells could traffic to the CNS. For patient 1, no CAR T-cells were detectable in the CSF during the 1st and 2nd months after CAR T-cell treatment; however, CAR T-cells were detectable in the CSF after PD when tumor invasion of the meninges occurred (Figure 1D, Supplemental Figure 2A). For patient 2, lymphoma cells were not detectable in the CSF at the 4th month after therapy, at which point the CAR T-cells in the CSF also disappeared. However, CAR T-cells could be detected again in CSF at the 8th month when disease relapsed. CAR T-cells percentage in lymphocytes was 88.77%. Furthermore, there were 40,000 copies/μg of the lentivirus for CAR19, but 0 for the CAR22 lentivirus at the 9th month (Figures 1C,D). Lymphoma cells from tissue biopsy were still CD19- and CD22-positive after disease recurrence in patient 2, and a small number of CD3^+^ T cells were observed to infiltrate into the tumor (Supplemental Figure 2B).

## Discussion

In our study, no patient experienced >2 grade CRS. Two patients experienced grade 1 or 4 CRES, although the CRES could be well-controlled by glucocorticoid and plasma exchange, and the neurologic toxicity-related symptoms were completely reversible. Our study has a similar incidence of neurotoxicity (2/5) to that of other reports (ranging from 21% to 64%) (9, 10, 13). CAR T-cell therapy for B-cell brain parenchymal lymphoma was well-tolerated, and the CRS and CRES were controllable without fatal events.

In our case series, all patients responded to CAR T-cell therapy, including three patients who achieved PR and two patients who achieved CR. The remission rate was similar to that in our previous research and other trials, but the PFS (PD in three patients at the 3rd month and relapse in one patient at the 8th month; the remaining patient was enrolled into another clinical trial) seemed to be substantially shorter than that from our previous report about systemic lymphoma (the median PFS: 9.9 months) (14). In a study of tisagenlecleucel therapy in secondary CNSL, three patients achieved CR; however, although only one relapsed case was observed, the other two patients were followed up for only 3 and 6 months. Longer observation times are needed (13).

Biopsy on one relapsed patient and flow cytometry of the CSF for the other PD patient were performed. We found some common features: (1) targeted antigens (CD22 and CD19) were still expressed on lymphoma cells of both patients; (2) CD3^+^ T cells were less abundant in the biopsied tissues; and (3) reboosted CAR T-cells were found in the CSF. In addition, persistent B cell aplasia (more than 6 months) and detectable CAR DNA copies indicated that CAR T-cells were not only present in the CSF and plasma but also functional. Therefore, we speculated that although CAR T-cell treatment is initially effective for CNS lymphoma, CAR T-cell therapy stress could change the tumor expression profiles of inflammatory factors and chemokines, which may influence the composition of inflammatory infiltrates in PCNSL. Interestingly, Nam et al. (18) reported that increased number of tumor-infiltrating CD204^+^ M2 macrophages was associated with poor clinical outcome in CNS-DLBCL, whereas increased number of CD68^+^ or indoleamine 2,3-dioxygenase+ cells was related to a favorable prognosis. In addition, PD-L1 is highly expressed in PCNSL and contributes to tumor immunosuppressive microenvironment (19, 20), which affects efficacy of CAR T-cell therapy. PD after CAR T-cell therapy may be caused by a mutative TME, a unique TME in the brain, such as activated microglia at the border zone of solid cerebral lymphomas, which is considered to be a reason for the lack of efficacy of CAR T-cell therapy for glioblastoma (21, 22).

Based on the longer follow-up observations and failure of continuous action of CAR T-cells in solid cerebral lymphoma, other consolidation treatments or combination therapy should be implemented when the best response is achieved after CAR T-cell infusion. For example, when patient 3 achieved CR, he was immediately enrolled in a second trial and remained in CR for more than 14 months, suggesting that effective CAR T-cell therapy combined with autologous HSCT (ASCT), especially high-dose chemotherapy with ASCT (HCT-ASCT), may be a better strategy for treatment. HSCs have been reported to be able to improve the TME (23). However, for patients not suitable for HSCT, PD1 blockers and/or adjuvant radiotherapy, which not only change the immune microenvironment but also enhance the efficacy of immunotherapy, might be alternative choice (24, 25).

In summary, although CAR T-cell therapy is effective for CNS DLBCL with an acceptable safety profile, our study suggests that CAR T-cell therapy should be combined with other treatments to help improve the TME of cerebral lymphoma. In addition, the five patients received heavily previous treatments and an early delivery of CAR T-cells may induce better results at least in some cases (26). Our study is limited by the small case series, and methods for improving TME after CAR T-cell therapy, which may prolong remission time, need to be further investigated.

## Ethics Statement

The studies involving human participants were reviewed and approved by Ethic Committee of Tongji Hospital. The patients/participants provided their written informed consent to participate in this study. Written informed consent was obtained from the individual(s) for the publication of any potentially identifiable images or data included in this article.

## Author Contributions

XZho and JZ designed the project. TL, YX, FM, DW, XZhu, LH, JW, and BX performed the clinical research. TL, XZho, and LZ analyzed the data and wrote the manuscript. LC, SL, XL, and YZ performed laboratory work for this study. LM analyzed the data. All authors contributed to the article and approved the submitted version.

## Conflict of Interest

The authors declare that the research was conducted in the absence of any commercial or financial relationships that could be construed as a potential conflict of interest.
