# Supplementary material for: CAR T-Cell Therapy Is Effective but Not Long-Lasting in B-Cell Lymphoma of the Brain
Source: Front Oncol. 2020 Aug 4;10:1306. doi: 10.3389/fonc.2020.01306 (PMC7438944; doi:10.3389/fonc.2020.01306)
Supplement: Supplementary file 1 [file Data_Sheet_1.DOCX]

**CAR T-cell therapy is effective but not long-lasting in B-cell lymphoma of the brain**

Tongjuan Li1*, Lei Zhao1*, Yuanyuan Zhang1, Yi Xiao1, Di Wang1, Liang Huang1, Liya Ma2, Liting Chen1, Songya Liu1, Xiaolu Long1, Fankai Meng1, Xiaojian Zhu1, Jia Wei1, Bin Xu1, Jianfeng Zhou1, Xiaoxi Zhou1#

1Department of Hematology, Tongji Hospital, Tongji Medical College, Huazhong University of Science and Technology, Wuhan, Hubei, China; 2Department of Radiology, Tongji Hospital, Tongji Medical College, Huazhong University of Science and Technology, Wuhan, Hubei, China.

*These authors contributed equally to this work.

^#^Corresponding authors.

**Correspondence**: Dr. Xiaoxi Zhou, Department of Hematology, Tongji Hospital, Tongji Medical College, Huazhong University of Science and Technology, 1095 Jiefang Avenue, Wuhan, Hubei 430030, China; Email: cello316@163.com.

**Method**

**T cell collection and generation of CD19 and CD22 CAR-T cells**

Apheresis products underwent CD19 and CD22 CAR-T cell manufacturing under protocols approved by the Ethic Committee of Tongji Hospital, Tongji Medical College, Huazhong University of Science and Technology, as previously described ([1](#_ENREF_1)). Briefly, after stimulated by anti-CD3/ anti-CD28 monoclonal antibody-coated magnetic beads, CD3+ T cells were separately transduced with different lentiviral vector encoding anti-CD19 or anti-CD22 scFv linked to CD28 and 4-1BB costimulatory domains and CD3-ζ signaling domain, and were expanded for 10 to 14 days in vitro. The transduction efficiency, endotoxin, mycoplasma and sterility of CAR T cells were tested prior to infusion.

## Reference

1. Li T, Zhang Y, Peng D, Mao X, Zhou X and Zhou J. A good response of refractory mantel cell lymphoma to haploidentical CAR T cell therapy after failure of autologous CAR T cell therapy. *J Immunother Cancer*. (2019) 7**:** 51 doi:10.1186/s40425-019-0529-9

**Supplementary table 1**: CAR T-cell infusion schedule.

| ID CAR | CD22 CAR T-cell | CD19 CAR T-cell |
| --- | --- | --- |
| Patient 1 | Day 0, 3 | Day 1 |
| Patient 2 | Day 0 | Day 1 |
| Patient 3 | Day 0-1 | Day 1-2 |
| Patient 4 | Day 0 | Day 1 |
| Patient 5 | Day 0 | Day 2 |

First CAR T-cell infusion day as day 0.

**Supplementary figure 1**

**A**


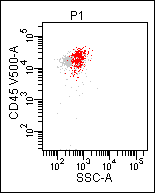

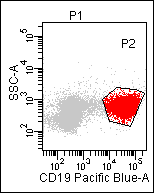

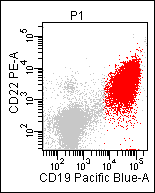


**B**

**
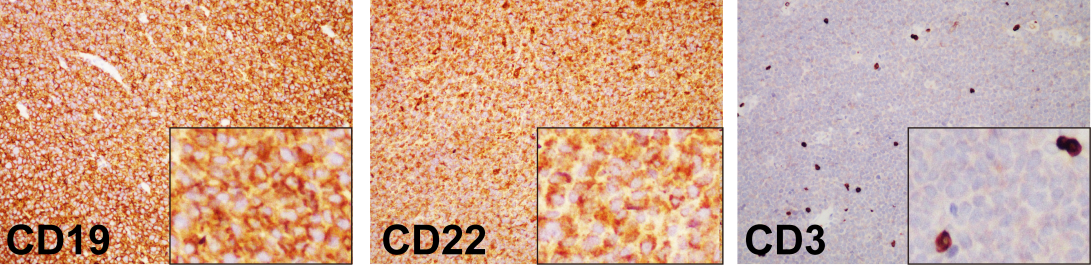
**

**Supplementary figure 1. Expression of CD19 and CD22 after disease recurrence.** (A) Lymphoma cells of patient 1 from CSF were stained with antibodies against CD45, CD19 or CD22 after disease recurrence and the expression of CD19 and CD22 in CD45+ cells were detected by flow cytometry. (B) Lymphoma cells of patient 2 from tissue biopsy in head were stained and detected for CD19, CD22 and CD3 by immunohistochemistry after disease recurrence.

**Supplementary figure 2**


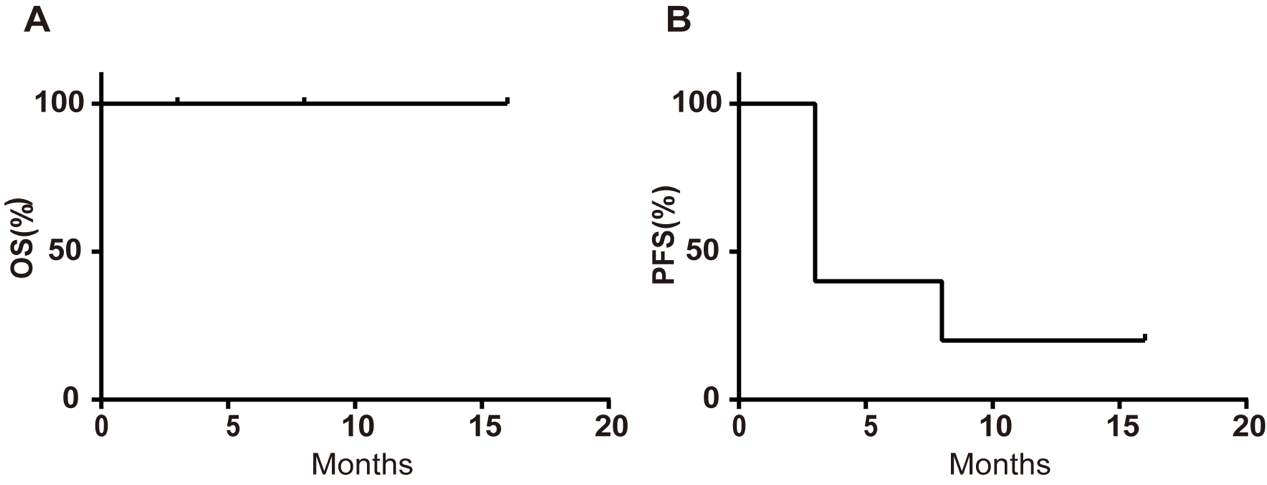


**Supplementary figure 2: The OS and PFS curves of the 5 patients.** (A) The overall survival was 100% in 6-16 months of follow-up. (B) The median progression free survival time was 3 months.
